# Body motion of choral singers

**DOI:** 10.3389/fpsyg.2023.1220904

**Published:** 2023-12-22

**Authors:** Sara D'Amario, Sten Ternström, Werner Goebl, Laura Bishop

**Affiliations:** ^1^Department of Music Acoustics, mdw – University of Music and Performing Arts Vienna, Vienna, Austria; ^2^RITMO Centre for Interdisciplinary Studies in Rhythm, Time and Motion, University of Oslo, Oslo, Norway; ^3^Department of Musicology, University of Oslo, Oslo, Norway; ^4^Division of Speech, Music, and Hearing, School of Electrical Engineering and Computer Science, KTH Royal Institute of Technology, Stockholm, Sweden

**Keywords:** togetherness, ensemble singing, motion capture, joint-actions, music perception, flow, voice matching

## Abstract

Recent investigations on music performances have shown the relevance of singers' body motion for pedagogical as well as performance purposes. However, little is known about how the perception of voice-matching or task complexity affects choristers' body motion during ensemble singing. This study focussed on the body motion of choral singers who perform in duo along with a pre-recorded tune presented over a loudspeaker. Specifically, we examined the effects of the perception of voice-matching, operationalized in terms of sound spectral envelope, and task complexity on choristers' body motion. Fifteen singers with advanced choral experience first manipulated the spectral components of a pre-recorded short tune composed for the study, by choosing the settings they felt most and least together with. Then, they performed the tune in unison (i.e., singing the same melody simultaneously) and in canon (i.e., singing the same melody but at a temporal delay) with the chosen filter settings. Motion data of the choristers' upper body and audio of the repeated performances were collected and analyzed. Results show that the settings perceived as least together relate to extreme differences between the spectral components of the sound. The singers' wrists and torso motion was more periodic, their upper body posture was more open, and their bodies were more distant from the music stand when singing in unison than in canon. These findings suggest that unison singing promotes an expressive-periodic motion of the upper body.

## 1 Introduction

Choir singing is a popular recreational activity, with proven benefits for choristers' social and mental wellbeing performing in face-to-face (Clift et al., 2010; Livesey and Camic, 2012; Judd and Pooley, 2014) and virtual (Daffern et al., 2021) choirs. Nevertheless, it requires blending several expressive parameters (such as intonation, timbre, timing and dynamics), and coordination of body movements, regardless of the level of expertise of the singers (Himberg and Thompson, 2009).

It is well-established that musicians' body motion is a core element of music performance. Research, primarily based on instrumental performances, has shown that musicians' body motion supports sound production and facilitates communication of expressive intentions and interactions with the co-performer(s) and the audience (Davidson, 2001; Jensenius et al., 2010). Certain body motions can also be unintentionally mimicked: emotional facial expressions whilst singing can lead viewers to produce subtle facial movements mimicking those of the musician (Livingstone et al., 2009).

In the context of singing, body movements of singers and conductors are fundamental aspects of choral music education and classical singing lessons. These movements represent powerful physical metaphors that can broaden singers' learning of musical ideas and enhance the singing experience during singing rehearsals (Apfelstadt, 1985; Peterson, 2000; Nafisi, 2013). A set of creative movements can be used in choir rehearsal to efficiently elicit certain musical concepts that might be less effectively communicated if taught only verbally. Hand gestures demonstrating phrase structure or dynamics can help perform the musical phrase and the dynamics contrast; changes from sitting to standing can facilitate the choir's perception and performance of diverse dynamics; walking, clapping or tapping the rhythm can support rhythm understanding (Peterson, 2000).

However, the literature regarding choristers' body motion is still in its infancy. Previous studies on small ensembles suggest that musicians' body motion can reflect the extent to which musicians are together (D'Amario et al., 2022) and features of the musical score (Palmer et al., 2019; D'Amario et al., 2023). This study focuses on ensemble singing and investigates if and how singers' body motion reflects the sensation of voice-matching with a co-performer and the complexity of the performance task, such as singing in unison (i.e., singing the same melody simultaneously) and canon (i.e., singing the same melody at a temporal delay related to a co-performer). In the next section, we reflected on (i) the choral sound factors that can affect singing preferences and how body motion can relate to (ii) the musicians' sensation of voice-matching, (iii) the complexity of the singing task, and (iv) certain sound parameters. We conclude by posing some hypotheses and drawing some predictions for this study.

### 1.1 Singers' preferences of choral sound factors

Singers agree, to some extent, on their preferences for several sound factors, which can be objectively measured. Choir singers and listeners prefer intentional intra-section singer configurations (in which choristers stand next to each other based on their timbral and acoustic compatibility) to random configurations, because of higher blend and tone quality and better ability to hear self and others in the intentional configurations (Gilliam, 2020). Soprano, alto, tenor, and bass (SATB) choristers prefer spread configurations between singers to close configurations, as spread spacing was found to increase hearing of the self and the ensemble and also contribute to a better choral sound compared to close configurations (Daugherty et al., 2012, 2019). The preferences of experienced singers for pitch scatter in unison choir sound were also explored. Results show that most listeners prefer a 0 level of pitch dispersion (i.e., same mean fundamental frequency between voices), and they would tolerate a 14 cents standard deviation in fundamental frequency (Ternström, 1993).

Singers might also have preferences for how their sound spectral components match those of the co-performer. Evidence from barbershop quartet performances demonstrates that singers spread their formant frequencies rather than align them (Kalin, 2005). This suggests that singers probably control their voice spectrum to blend their voices with the ensemble (Kalin, 2005). Preferences for voice-matching might reflect musicians' experiences of togetherness, i.e., feelings of being and acting together during joint music performance (Hart et al., 2014; Noy et al., 2015; D'Amario et al., 2022). These togetherness feelings are particularly relevant to choirs, in which choristers are required to blend their pitch, intensity, vibrato, timbre and timing with those of the co-performers. However, singers' preferences for voice-matching and their perception of togetherness in choirs lack a thorough investigation. The present study investigates whether singers' sensation of voice-matching with a co-performer depends on the spectral envelope of the sound. In order to control and manipulate the spectral components of the co-performer, we used a singer-loudspeaker paradigm in which choristers performed along to a pre-recorded tune presented via a loudspeaker.

### 1.2 Body motion and interpersonal interactions

Research on interpersonal interactions during joint action activities, such as dancing and ensemble playing, has investigated the relationship between body movements' synchrony and judgments of togetherness, pro-sociality and aesthetic experiences of the performing arts. The perceived and performed synchrony of a group of dancers can positively correlate with an audience's perceived enjoyment (an index of their aesthetic appreciation), depending on the dances performed (Vicary et al., 2017). This suggests that judgements and performance of movement synchrony can relate to the aesthetic experience of the performance. Similarity in body movements in collective dance improvisations can correlate with dancers' enjoyment (Himberg et al., 2018). Manipulated interpersonal movement synchrony also correlates with pro-sociality. It has been found that patterns of distributed coordination emerging in large group dancing movements predict pro-social effects and group bonding (von Zimmermann et al., 2018). Overall, dancing studies show that moving together with others can bring aesthetic pleasure to the participants and audience members, and increase a sense of group affiliation.

In the context of music ensemble performances, body motion can also contribute to the perception of interpersonal interactions. Similarity in body movements coordination in non-pulsed duo improvisations can relate to judgments of interactions bouts (Eerola et al., 2018). The strength of synchronicity in common periodic movements of co-performers can positively relate to ratings of perceived synchrony (Jakubowski et al., 2020). In small ensembles, certain measurable patterns of body motion, such as similarity in arm and chest motion, can contribute to the judgments of togetherness, i.e., the extent to which musicians were together as judged by audience members (D'Amario et al., 2022). Body motion might also reflect togetherness sensation that musicians feel with a co-performer during ensemble playing, as they can be more open to communication. Behavioral studies suggest that posture openness can relates to communication intentions (McGinley et al., 1975; Grachten et al., 2010). It was found that singers' body was more open when performing in the presence rather than the absence of an audience (Grachten et al., 2010); and, that an open body posture can facilitate the communicator's intention to change opinion in others (McGinley et al., 1975). It is still unclear whether choristers' body posture reflects the sensation of voice-matching perceived in singing ensembles. Furthermore, the extent to which musicians experience togetherness could affect their peripersonal space, i.e., the region of space immediately surrounding our body. A recent study on jazz duo performances suggests that music performances might affect the perception of the space between performers by prompting them to withdraw from their partner under uncooperative conditions (Dell'Anna et al., 2020). However, the relationship between body position and voice-matching has not been investigated yet. The current study investigates if and how the sensation of voice-matching, which can be seen as an aspect of musical togetherness, affects musicians' body openness and peripersonal space.

Furthermore, social interactions in ensembles are multimodal processes involving different sensory modalities (Keller and Appel, 2010), featuring continuous adaptations with the co-performers (Timmers et al., 2014), and skilful body co-regulations (Leman, 2007) within and between performers. Recent attempts to investigate social interactions in virtual reality suggest that real-time interactions between performers in small ensembles, which are mediated by embodied avatars, might induce strong feelings of social presence; however, the interactions between a real musician and a computer-controlled agent, highly affecting co-performer responsiveness, might negatively impact the quality of the subjective experience (Van Kerrebroeck et al., 2021). In the current study, we implemented a singing-loudspeaker paradigm to investigate if and how voice-matching preferences impact body motion. This paradigm allowed the participants to manipulate the co-singer voice and identify the spectrum envelope they felt most and least together with at the expense of the co-performers' real presence and the associated continuous response. Although this paradigm does not allow the thorough study of social presence and togetherness feelings, this set-up enables the study of how a specific aspect of musical togetherness (i.e., voice-matching) contributes to choristers' body motion.

### 1.3 The effects of task complexity on musicians' body motion

Differences in the auditory feedback from the self or the co-performer can influence the accuracy of the music performance. Pfordresher (2005) and Pfordresher and Palmer (2006) found that mismatches between the production of pitch events and the corresponding auditory feedback disrupted the accuracy of the performance, measured in terms of pitch errors. The relationship between the auditory feedback produced by two performers affects the temporal coordination of duo performances. Zamm et al. (2015) found that onset synchronization was tighter, mutual adaption higher and tempi faster when piano duos performed the same melody in unison than in canon, suggesting that unison playing was easier. These findings were also somewhat corroborated by a later study, investigating acoustics and head coordination in singing duo performances, and observing slower tempi (but no differences in overall asynchrony) when singing with an offset than in unison (Palmer et al., 2019). Interestingly, the authors also found that these different singing productions affected head movements in the Follower (i.e., co-performer entering at a temporal delay): the Follower exhibited higher variability of head movements, changed head orientation more away from the co-performer and bobbed the head more when singing the same melody at a temporal offset than in unison. Overall, these results suggest that task complexity can affect performance accuracy and musicians' head movements. When a music stand is available, musicians might also stand closer to the music stand to improve sight-reading with increased task complexity. This study analyses changes in singers' head motion and distance from their music stand, during unison and canon singing.

Furthermore, task demands might also impact singers' hand motion, often free from external constraints such as holding an instrument or a music score. Similarly to the above findings analyzing head motion during canon and unison performances (Palmer et al., 2019), the disruption of a singer's own auditory feedback that occurs during canon singing might induce a disruption in the hand motion as well. The periodicity of hand motion during ensemble singing could reflect the ease of unison singing compared with the increased complexity of a canon performance. We tested the hypothesis that unison performances feature higher hand motion periodicity than canon singing by analyzing hand motion while a singer performed the same tune in unison and in canon.

### 1.4 Sound parameters

A line of research investigated the relationship between sound and motion. Eitan and Granot (2006) observed how changes in musical parameters related to images of motion; listeners in the study were asked to associate melodic stimuli with imagined motions of a human character and then describe the type, direction, pace-change and forces of these motions. The authors found that listeners map musical parameters to kinetic features: decreases in one parameter (e.g., pitch descents, ritardandi, and diminuendi) were associated with spatial descents, whilst intensifications of musical features (e.g., pitch rising, accelerandi, and crescendi) were paired with increases in speed rather than the ascent. Importantly, they revealed this relationship's complex and multifaceted nature: musical parameters were simultaneously associated with multiple motion parameters. Motion can also be correlated with the musical structures of the piece, for example in line with the *ritardandi* (Repp, 1992). So-called sound-tracing experiments, focused on the listeners' spontaneous gestural renderings of sound, have further analyzed listeners' immediate association between music and motion (Godøy et al., 2006; Nymoen et al., 2010, 2011, 2013). Interestingly, a strong positive correlation was found between vertical hand position and pitch, in listeners instructed to move their hands in the air as if they were creating the sound themselves whilst listening to a set of short sounds, with manipulated pitch, timbral and dynamic contours (Nymoen et al., 2011, 2013). These results are in line with findings based on mental imagery (Eitan and Granot, 2006). This association could be understood through learned metaphors: the pitch is explained as a vertical dimension in line with the order of the notes in a musical staff (Nymoen et al., 2013).

Among music performers, the analysis of spontaneous gestural responses to music in singers whilst performing is particularly valuable, since singers' hand motion is not constrained by a musical instrument. Certain singers' arm gestures have been found to be related to specific acoustical measures of singing, such as intonation and timbre. A low, circular hand gesture, as well as an arched hand gesture, were found to be related to singing timbre, as formant frequencies were lower when singers performed with gestures than without (Brunkan, 2015). A low, circular arm gesture can also impact tuning, depending on the piece being performed and the vowels analyzed. Intonation of the /u/ vowel of the word “you”, analyzed in 49 singers performing the final phrase of “Happy birthday to you”, was found to be closer to the target pitch when singing was paired with a low circular arm gesture than without (Brunkan, 2016). However, a low, circular gesture did not impact intonation when performing “Over the rainbow” (Brunkan, 2015; Brunkan and Bowers, 2021). In addition to the circular arm gesture, a pointing arm gesture can also affect intonation. Brunkan (2015) and Brunkan and Bowers (2021) found that singers performing “Singin' in the rain” were more in tune when singing with a pointing gesture than with no arm movements. A coupling between gesture and tuning was also found in Karnatak music performances, i.e., a south Indian music performance featuring multimodal expression (Pearson and Pouw, 2022). The authors also found that the coupling between wrist gestures and tuning was stronger than between gestures and voice amplitude envelope.

Overall, these studies suggest that certain arm gestures can affect intonation of certain sustained vowels; however, the specific direction of this gesture during entire music performances remains unclear. We further investigated these aspects, by testing the hypothesis that the continuous vertical motion of the right and left hands during singing performances are positively related to intonation tracking.

### 1.5 The current study

The current study investigates the effects of the perception of voice-matching and the complexity of the task on choristers' body motion whilst performing along with a pre-recorded tune presented over a loudspeaker. Voice-matching is conceptualized as a musical parameter contributing to togetherness feelings, i.e., feelings of being and acting together with a co-performer when performing in singing ensembles. The pre-recorded tune was chosen to replace a live singer's performance and allow manipulation of the tune they were listening to and singing along to.

We were first interested in analyzing the sensation of voice-matching with a co-performer in relation to the spectral envelope of the co-performer, which we addressed by asking singers to manipulate the filter settings. Based on barbershop quartet performances (Kalin, 2005), we hypothesized (hypothesis, H hereafter) that the long-term specrum envelope of the stimulus sound, as assessed over an entire song, has an effect on the sensation of voice match with a co-performer (H1). We then tested the hypothesis that singing along to recordings they felt most or not together with impacts body motion (H2). Based on recent investigations on togetherness judgment in small ensembles (D'Amario et al., 2022), we hypothesized that singers, whose body is not constrained by holding a musical instrument, stay further apart from the loudspeaker (representing a co-performer) when singing along to recordings featuring the least together setting rather than recordings they felt most together with (H2.1). Moving away from the least together setting could also be considered an avoidance behavior. Based on behavioral studies (McGinley et al., 1975; Grachten et al., 2010), we also conjectured that singers' upper body posture is more open (i.e., with head, shoulders and elbow more distant from the chest when singing along with the most than the least together setting), as they might be more open to communicating and interacting with the co-performer when singing with the most together setting (H2.2).

Furthermore, we hypothesized that task demands (i.e., singing the same tune in unison or canon) affect body motion (H3), based on studies suggesting the effects of task demands on interpersonal synchronization and body motion during ensemble performances (Zamm et al., 2015; Palmer et al., 2019). Specifically, we hypothesized that, when singing in canon, singers display higher quantity of body motion (in line with an overall increase of energy in the performance, H3.1), stand closer to the music stand (to follow better the score during the most challenging task, H3.2) and exhibit lower periodicity in wrist movements [based on disruptive aspects of the auditory feedback (Pfordresher and Palmer, 2006), H3.3]. In addition, we were also interested in analyzing the relationship between tuning and singers' wrist motion. In line with previous studies on singers' hand gestures (Brunkan, 2015; Brunkan and Bowers, 2021), we hypothesized that the vertical displacement of the wrists is positively correlated with intonation (H4), as singers would use the wrists' vertical motion to support tuning of higher notes, which is anecdotally reported as being more difficult than for the lower notes.

## 2 Methods

### 2.1 Design

The research study comprised a 2 (task mode: perception and production) × 2 (task complexity: unison and canon) × 2 (voice-matching: most and not at all together) × 2 (takes: take 1 and take 2; i.e., repetitions of the same task complexity / voice-matching condition) × 2 (blocks: block 1 and block 2; i.e., repetitions of both take 1 and take 2) design. The perception task focused on singers' voice-matching perception; the production task centered on singers' performance based on the voice-matching preferences collected during the perception task. Overall, this design provided a total of 16 perception trials per participant for the perception task, and 16 production trials per participant for the production task.

The order of task mode (i.e., perception and production) and block (i.e., block 1 and block 2) was fixed. The order of conditions in the perception task was fixed within block as follows: (i) first, two consecutive repetitions in unison then in canon of the least together setting; second, (ii) two consecutive repetitions in unison then canon of the most together setting. Within each block of the perception task, the least together setting was presented before the most together setting as the former can be seen as a practice condition to honing the participant's listening. Table 1 shows the design of the perception task. The order of conditions in the production task was randomized within part. Twenty-five production trials (of the 240 collected during the perception task) were excluded from the analysis because singers did not perform the piece according to the researchers' requirements (i.e., singing in canon or unison) or temporarily stopped singing. The corresponding perceptual trials were also excluded from the analysis.

**Table 1:** Design of the perceptual task, showing block, take and trial number as well as the voice matching and task complexity levels.

**Block**	**Take**	**Voice-matching**	**Task complexity**	**Trial**
**1**				
	1	Least together	Unison	1
	2	Least together	Unison	2
	1	Least together	Canon	3
	2	Least together	Canon	4
	1	Most together	Unison	5
	2	Most together	Unison	6
	1	Most together	Canon	7
	2	Most together	Canon	8
**2**				
	1	Least together	Unison	9
	2	Least together	Unison	10
	1	Least together	Canon	11
	2	Least together	Canon	12
	1	Most together	Unison	13
	2	Most together	Unison	14
	1	Most together	Canon	15
	2	Most together	Canon	16

### 2.2 Experimental set-up and apparatus

**Figure 1:**
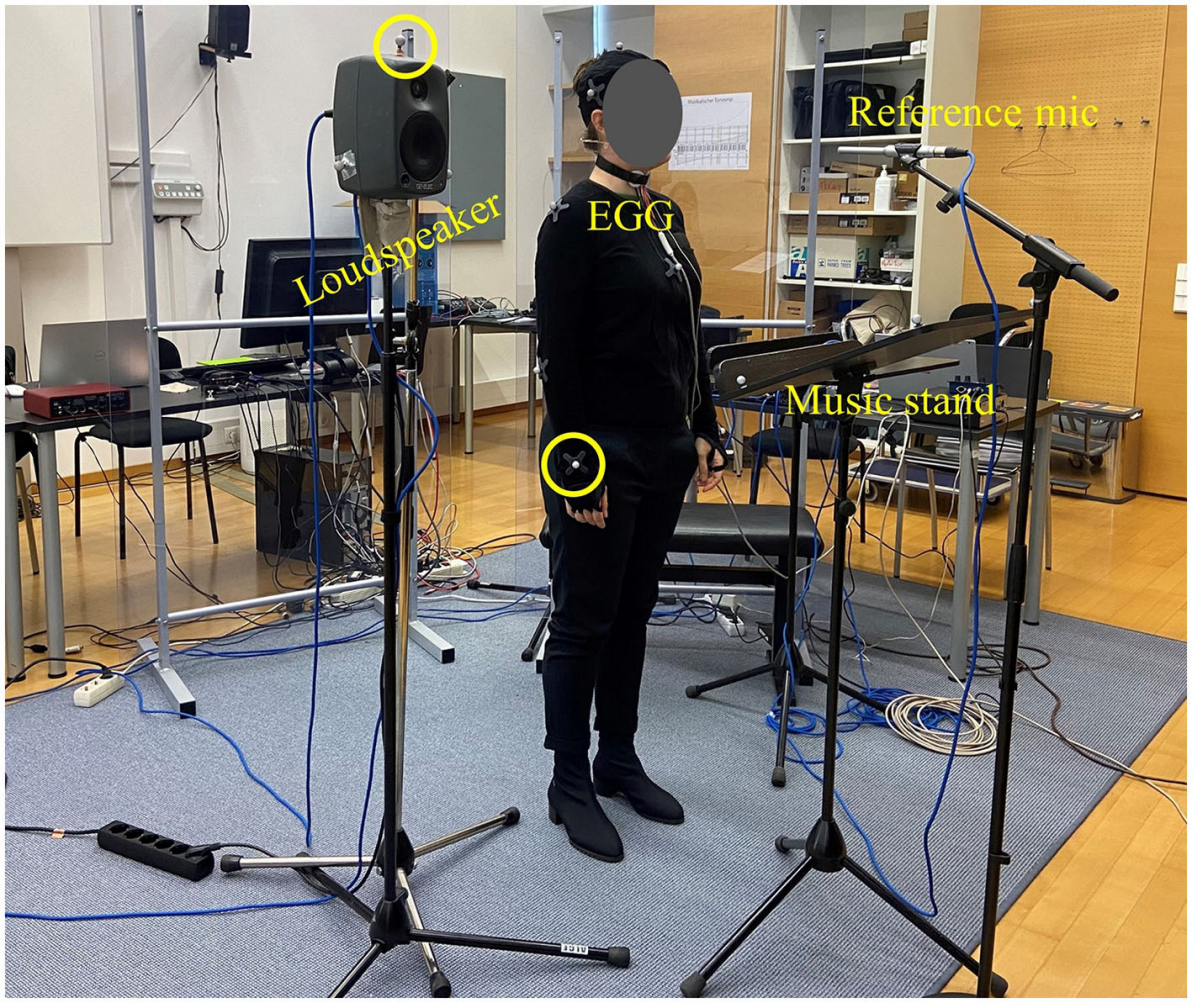
Example of the experimental set-up, showing some of the motion capture markers placed on the singer's right wrist and the loudspeaker (circled). The figure also displays the electroglottography (EGG) electrodes on the singer's neck as well as the reference microphone and the music stand placed in front of the singer.

The experiment was conducted in a large multipurpose seminar room at the Department of Music Acoustics of mdw – University of Music and Performing Arts Vienna, equipped with a motion capture system suspended from the ceiling. The participant stood near the center of the motion capture rig, whilst two researchers sat at their desks behind the participant, to record audio and motion capture data. A small high-quality active studio loudspeaker was stand-mounted 0.7 m to the singer's right (Genelec model 8020C, www.genelec.com, with its built-in tone controls set to flat). The sounds of the pre-recorded performances were played at a sound level calibrated such that the loudspeaker radiated the same acoustic power as had the singer who pre-recorded the stimulus sound. The participant and the loudspeaker faced a wall with acoustical drapes at 2.7 m distance, facing away from the researchers' desks. A music stand holding the score was placed in front of the singer, angled so as to avoid acoustic reflection back to the singer. A computer screen 2 m in front of the singer, also angled away, displayed visual prompts and instructions from the computer to the participant. Figure 1 shows an example of the experimental setup. For the perception task a small MIDI controller device (Native Instruments, model 4CONTROL) was placed in front of the participant; on this device, two (out of four) endless, unmarked rotary knobs were used by the participant to control the gains (±15 dB) of two parametric filters centered on 2.7 and 6.2 kHz. The 2.7 kHz frequency band corresponds to the “singer's formant (cluster)”. When the level in this band is very high, the voice timbre is perceived as “piercing” or “projecting” or even “harsh”. The 6.2 kHz frequency band corresponds to a region in which a moderately raised level gives an impression of “clarity”, “proximity”, or “airiness”. When the level in this band is lowered, the voice sounds occluded, as if facing away. An on-screen yellow indicator would light up if a participant repeatedly tried to change the gain in either band beyond ±15 dB, to indicate that further change in that direction would be futile. Also, one push button (out of two) on the controller was used to signal “Next”. All movable flat surfaces (screens, music stand, display) were angled so as to avoid direct reflection paths from the singer's mouth to the microphones. As a pandemic precaution, plexiglass screens separated the experimenter tables from the participant.

A 12-camera (Prime 13) OptiTrack motion capture system was used to record the participants' body motion at a sampling rate of 240 Hz. Singers' body motion data consisted of trajectories from 12 reflective markers placed on the head and upper body: three markers on the head, two on the back, one per shoulder, arm, and wrist, and one on the chest. Three additional markers were placed on the loudspeaker (one on top and two on the side near the loudspeaker) and five more on the music stand (one in each corner and one on the top of the stand). These additional markers were used to investigate the position of the singers with respect to the loudspeaker and the music stand.

Participants also wore electroglottography (EGG) electrodes (Glottal Enterprises model EG2-PCX—using the analog output) placed on the neck, either side of the thyroid cartilage. EGG is widely used to analyse the singing voice (D'Amario and Daffern, 2017; Herbst, 2020) and allows individual fundamental frequency analysis for each singer based on vocal fold activity rather than microphone recordings. Therefore, it was used in this study for tuning analysis without cross-talk. In addition, a ‘backstage' microphone (Neumann KM A P48) was placed near the experimenters' desk recording motion data, to synchronize motion and audio recordings. Audio recordings were synchronized with OptiTrack recordings using an audiovisual signal produced by a film clapboard, marked with reflective markers. The clapper was placed in view of the motion capture cameras and close to the “backstage” microphone. The clapboard was struck at beginning of each part, and all recordings were synchronized retrospectively to this point. At the start of each trial, the control program also issued a sequence of N clicks on the same channel, to facilitate the localization of trial N on the motion capture recordings.

Furthermore, four additional microphones were used, including a reference microphone in front of the singer, a headset microphone near the singer's mouth, and two binaural microphones just in front of either ear. These microphones acquired signals for an acoustic corollary study that is out of scope here; it will be reported elsewhere.

MIDI and audio input and output data (excluding that of the backstage microphone) were routed through a multichannel, multipurpose digital audio interface (RME model UFX II) to the computer (Microsoft model Surface Book 2, Windows 10 Enterprise). Audio from the backstage microphone fed into a multi-channel audio interface (Focusrite Scarlett 18i8), recorded using a digital audio workstation (Ableton Live) at a sampling frequency of 44.1 kHz and 32-bit depth, using a second PC. Experimental instructions to participants, stimulus presentations and audio recordings were run automatically by custom programs written in SuperCollider (v 3.12.1, http://supercollider.github.io).

### 2.3 Participants

Seventeen participants (age *M* = 31.2 years old, *SD* = 11.2 years; 6 women, 11 men) took part in the study. Fifteen of them took part in the perception and production tasks of the experiment; these were semi-professional singers, singing students at mdw – University of Music and Performing Arts Vienna and/or choral singers of local choirs at the time of the experiment. They reported having on average 9.5 years of formal training (*SD* = 4.3 years) and practicing on average 1.5 h per day (*SD* = 0.7 h). All participants self-reported normal hearing, and three self-reported perfect pitch. They received a token compensation of 30 Euros. The Ethics Committee at mdw – University of Music and Performing Arts Vienna approved the procedures of this study (reference EK Nr: 05/2020).

In addition to the above 15 participants completing the perception and production tasks, two participants took part in the study as pre-recorded co-performers and their singing recording were presented through a loudspeaker. These were professional singers, with advanced choral experience. They had on average 7.5 years of formal singing training.

The sample size was set in line with the relevant literature (Livingstone and Palmer, 2016). Participants were recruited on a voluntary basis through advertisements on mdw social media and semi-professional choirs.

### 2.4 Stimulus

#### 2.4.1 Music score

**Figure 2:**
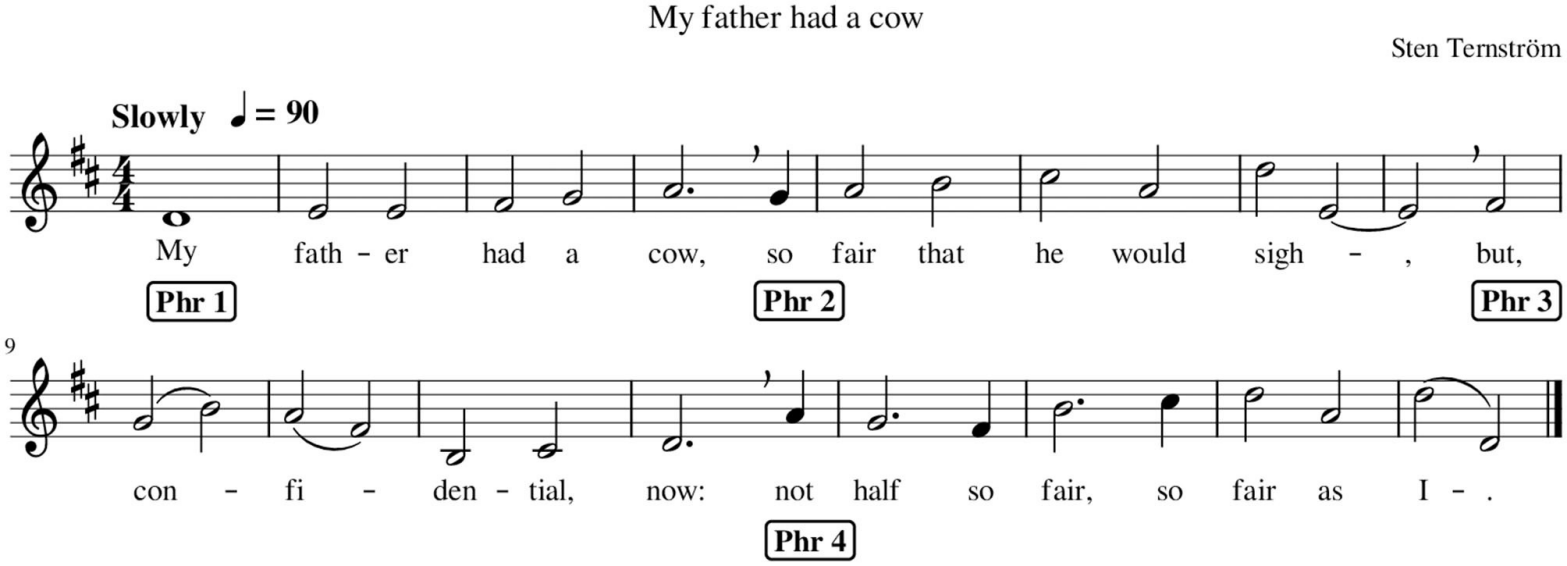
Tune composed for the current investigation, displaying the beginning of its four musical phrases, as marked in the score.

A short 16-bar piece (as shown in Figure 2) was composed for the study by the second author (ST), such that the piece was simple enough to be learned quickly and could function as a two-part canon at two bars offset. Many long sustained notes were employed in the piece, to facilitate intonation stability throughout. The lyrics, in English, were written to contain many open vowels and few sequences of multiple consonants. None of the participants had any difficulties with pronunciation.

#### 2.4.2 Co-performer stimulus pre-recording

In order to create the stimulus recordings to be presented to the participants completing the perception and productions tasks, two professional musicians came to our multi-purpose seminar room before beginning the experiment and practiced the tune for about 15 min, then performed the piece until they reached satisfaction. Singers were instructed to perform as they would normally do in the choir, with a strict tempo and a limited vibrato. During the practice trials, four metronome beats at 90 beats per minute cued the tempo; after that, the metronome was turned off. Audio recordings of repeated performances were collected. The recording considered the best choral performance was chosen as the pre-recorded stimulus for the current study, so the participants could sing in duo along with it. The singers judged and chose their best performance.

### 2.5 Procedure

Participants were invited to take part in a single session. First, participants received spoken and written explanations of the research project and the tasks, then they gave written consent to participate in the study and filled in a background questionnaire regarding their music experience. They were also asked whether they had ever experienced optimal voice matching when singing with a singer in a choir or small ensemble; if yes, they were invited to describe their feelings at that moment.

Having practiced the score in the laboratory, participants first made a baseline recording without a co-performer stimulus. This was recorded using a reference microphone at 0.3 m in front of the participant, in addition to the headset and binaural microphones, in order to determine the relative frequency responses of the latter. This aspect will be reported in a companion study, as the acoustics analysis is out of the scope of the current study.

Then, the perception task was presented. Singers listened to and sang the song together with a set of recordings, in each trial modifying the stimulus sound using the two rotary knobs until they heard the sound they felt “most” or “not at all” together with, according to text prompts provided on a screen. The participants were not told what the knobs did; they had to hear it for themselves. Participants were free to move “as they might do in a choir”. Headset and binaural microphones were fixed to the singer, so recording quality was not affected if they drifted away from the starting point. The initial filter gain settings were invisibly randomized at the start of each trial. The stimulus song was looped continuously until the participant pressed “Next”. The filter settings ultimately chosen by the participant for each trial were saved.

Ultimately, the performance task was presented, and participants sang the piece, performing in duo with the pre-recorded performance processed using the filter settings they had chosen in the perception task. Participants were asked to perform along to loudspeaker pretending to be on stage and performing with a choral singer. Participants were left free to sing by memory or look at the score (placed on the music stand in front of them) as best for them. Therefore, no particular instructions regarding looking at the music score were given. Pre-recorded stimuli were gender-matched with the participants, since we needed to control the spectral differences between stimulus and performer as closely as possible. The stimulus song was played only once per trial, without looping. Singers took a 5-min rest between the two tasks and also between the two blocks during the performance task.

### 2.6 Analysis

To investigate singers' body motion in relation to voice-matching perception and task complexity, the following metrics were computed:

- Magnitude of the gains of mid and high-band
- Quantity of motion (*QoM*) measuring the overall energy of the performance
- Singer's upper body posture
- Singer's distance from the loudspeaker and the music stand
- Periodicity of the singer's head and wrist motion
- Wrist's vertical motion

To assess whether preferences for spectral components of the sound change based on the perception of voice match, we computed the magnitude of the settings of the mid and high-band filters that singers chose during the perception task. Magnitude was operationalized by taking the square root of the sum of the squares of the chosen filter gains in dB. This magnitude is the length of a vector and represents the distance from the origin (0,0) dB to the endpoint of the vector.

To compute the metrics related to singers' movements, motion capture data were first subject to pre-processing: data of all markers were smoothed and velocity derived using a Savitzky-Golay filter (polynomial order 3, window size 25), through the “prospectr” package (Stevens and Ramirez-Lopez, 2021) in R (R Core Team, 2013). The speed was then calculated as the Euclidean norm of the three-dimensional (3D) velocity data.

Then, the total quantity of motion (*QoM*) was calculated as the sum per second of the Euclidean norm of 3D velocity values across all markers for each singer and repeated performance. Then, the *QoM* was averaged across time stamps within phrases. This step produced four *QoM* values (one per phrase) for each singer and repeated performance. Because *QoM* data were not normally distributed, they were log-transformed before the analysis.

Regarding the singers' upper body posture, this was operationalized as the summing of the 3D distance between the chest and the front head, and between the chest and all the peripheral joints under investigation (i.e., left and right shoulder and elbow). In other words, large distances between body parts would suggest a more open body posture, while small distances would suggest a more contracted posture. The distance between the singer and the loudspeaker was computed as the 3D distance between the front head marker and the marker placed on the loudspeaker near the singer. Similarly, the distance between the singer and the music stand was computed as the 3D distance between the front head marker and the marker placed at the bottom left corner of the music stand. The vertical motion of the wrists was computed based on the Y-axis position data, perpendicular to the ground plane. These metrics were averaged within phrases.

**Figure 3:**
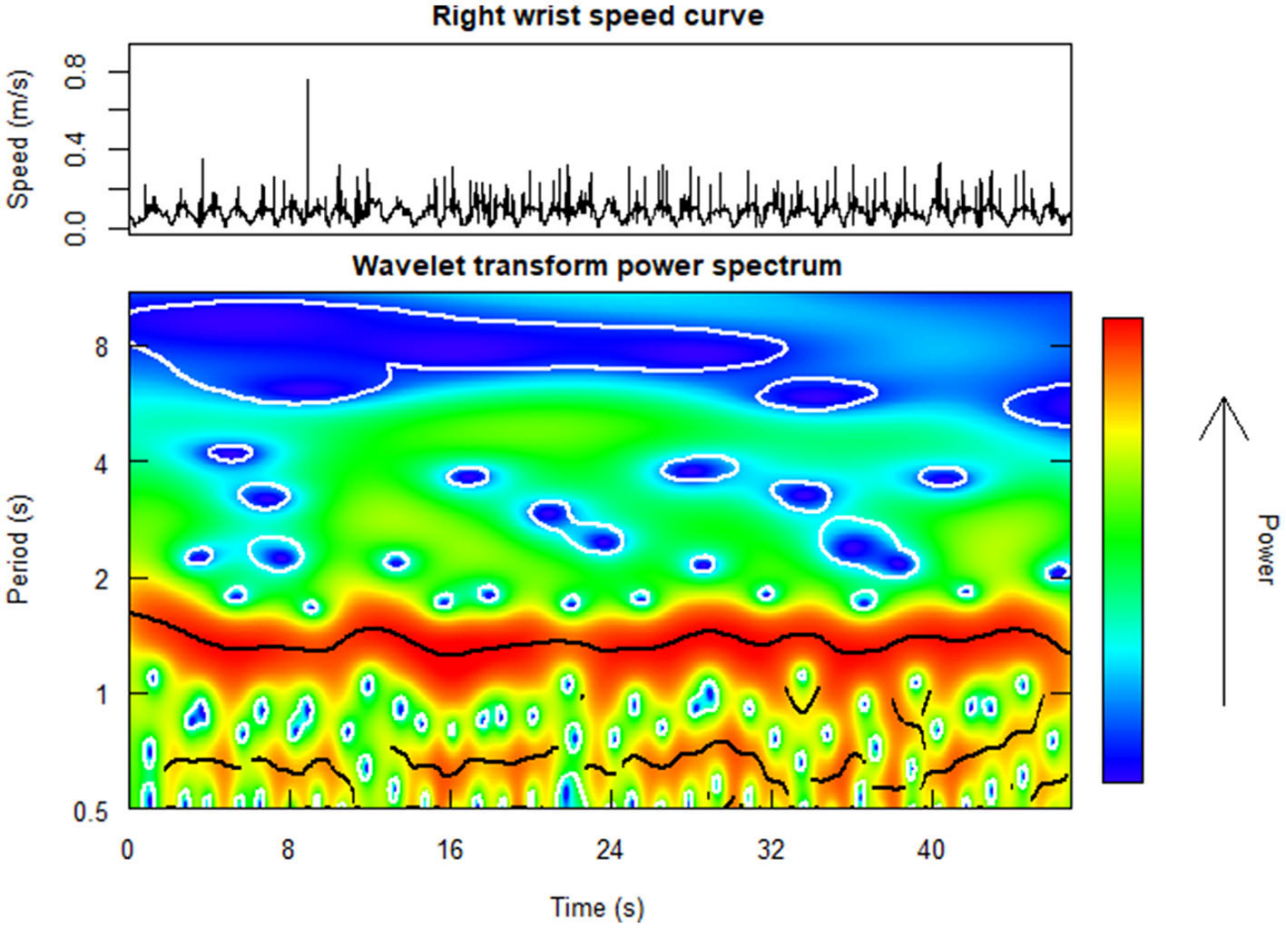
Example heat plot of the wavelet transform (WT) power spectrum (at the **bottom**) as computed from the right wrist speed curve (at the **top**) for a chorister performing the short tune in unison along with a pre-recorded performance of the same piece. The warmer the map, the more periodic the signal is.

The periodicity in the singers' front head and wrist motion was computed by extracting the power of the wavelet transforms (*WT*) of the speed curves of chosen markers (i.e., front head, left and right wrist), for each repeated performance and singer, as shown in Figure 3. *WT* were extracted using the R package “WaveletComp” (Roesch and Schmidbauer, 2018) with the complex-valued Morlet wavelet as mother wavelet. The range of periods to be considered was set in line with the phrase structure and the tempo of the piece, and ranged from about 1 beat (mean duration = 0.667 s) to 4 bars (mean duration = 10.36 s). Thus, the range of the *WT* extraction was from 0.5 s to 11 s. Within this broadband, the average *WT* power was computed within a narrow band centered around the maximum power spectrum with a width of ± 1 beat. Ultimately, *WT* data were averaged across timestamps within phrases; this produced a list of four *WT* values (one per phrase) for each performance and singer.

A linear mixed model was implemented to analyse the effects of voice match and task complexity (fixed effects) on the magnitude of the spectral components (response variable). Block, take, and trial were entered in the model as fully crossed random effects. In addition, two sets of linear mixed models were then implemented to investigate whether the above body motion metrics were predicted by the following explanatory variables:

- Block, take, trial, and phrase, and
- Voice match (i.e., most and not at all together) and task complexity (i.e., singing in unison vs. canon).

For each body metric, two models were run. The first model included block, take, trial, and phrase as fixed effects. Only significant effects were retained for the second model, which additionally tested the effects of voice match and task complexity. For example, for *QoM*, the first model showed significant effects of take, trial, and phrase, but no significant effect of block. *QoM* was therefore averaged across blocks, and the second model tested the effects of voice match, task complexity, take, trial, and phrase. These models were implemented via the residual maximum likelihood (REML) in R using the lme4 package version 1.1-27.1 (Bates et al., 2015); *p*-values for fixed effects were calculated using Satterthwaite approximation through the lmerTest package version 3.1-3 (Kuznetsova et al., 2017).

In addition, to investigate whether the wrists' motion was affected by tuning, the fundamental frequency (*f*_o_) estimates were extracted from the electroglottography (EGG) recordings using the “PraatR” package in R (Albin, 2014). The tuning analysis relied on the EGG recordings collected during the experiment, as EGG allows estimating the individual contribution of the singer without cross-talk from the co-performer (i.e., loudspeaker). The *f*_o_ trackings were extracted in 10 ms time steps in the frequency band from 70 to 660 Hz. This frequency band was chosen to cover the male and female expected voice range profile featuring this piece. Tuning data were then standardized (i.e., scaled and centered) based on singers' gender to account for differences in voice range between males and females. The time-series *f*_o_ estimates of each performance and singer were then averaged per decisecond; similarly, wrists' vertical position data presented above were also averaged per decisecond in order to have the two data set sampled at the same frequency (i.e., 10 Hz).

Eventually, to investigate the effects of tuning on the wrist's vertical displacement, four linear mixed models (i.e., one for each combination of block [block 1 and block 2] and task complexity [unison and canon]) per wrist were implemented using the glmmTMB package (Brooks et al., 2017) in R. In each model, times were entered within stimuli with the Ornstein–Uhlenbeck covariance structure, which can handle unevenly spaced temporal autocorrelation. Tuning data included short pauses due to the singers breathing for tone production or in line with the score requirements (e.g., see Figure 2 bar n. 4) or due to own errors (i.e., singers occasionally skipping a note). In each model, tuning data was entered as the response variable and the wrist's vertical position data as the independent variable; participant and trial number were entered as random effects.

Benjamini and Hochberg (1995)'s false discovery rate (FDR) correction was applied for multiple linear mixed models. The correction was based on a total of 81 *p*-values (i.e., 70 related to the 20 body motion models, 8 related to the 8 tuning models, and 3 related to the model testing singers' preferences of optimal matching) resulting from the analysis of the fixed effects, i.e., voice matching, task complexity, tuning, block, part, phrase number.

## 3 Results

### 3.1 Singers' preferences of optimal matching

The magnitude of the absolute gains in dB of the mid and high-band filters was predicted by voice match (β = 5.2, *SE* = 0.9;*t* = 5.7, *p* < 0.001), and the mean magnitude was significantly greater for the least together settings (*M* = 17.4, *SD* = 4.32) than for the settings chosen as all together (*M* = 12.9, *SD* = 5.42). The main effect of task complexity and the interaction effect between voice match and task complexity were non-significant.

Table 2 presents the raw responses to the questions: “Have you experienced optimal voice matching when singing with a singer in a choir or small ensemble? If yes, could you please describe that moment?”. Most of the participants (12 out 15) reported they experienced optimal matching in the past. During those recalled times, three of them felt as if everything flowed and was locked in so as to become one entity. Three of them focused on the positive emotions perceived, as they felt the moment was effortless, magical and rewarding.

**Table 2:** Raw responses (corrected for spelling mistakes) to the question regarding previous experiences of optimal voice matching.

**Have you experienced optimal voice matching?**	**If yes, could you please describe that moment?**
yes	n.a.
yes	When I got the feeling, everything flowed, and we got one.
yes	n.a.
no	n.a.
yes	All locked in! Sound amazing!
n.a.	n.a.
yes	Feeling as if we are one.
yes	It was a surprise, “a miracle”.
yes	Matching vibrato
yes	n.a.
yes	Matching in terms of voice color
n.a.	n.a.
yes	It makes me feel more confident, and I appreciate better the beauty of the sound.
yes	It felt effortless and easy.
yes	n.a.

### 3.2 Quantity of motion

**Figure 4:**
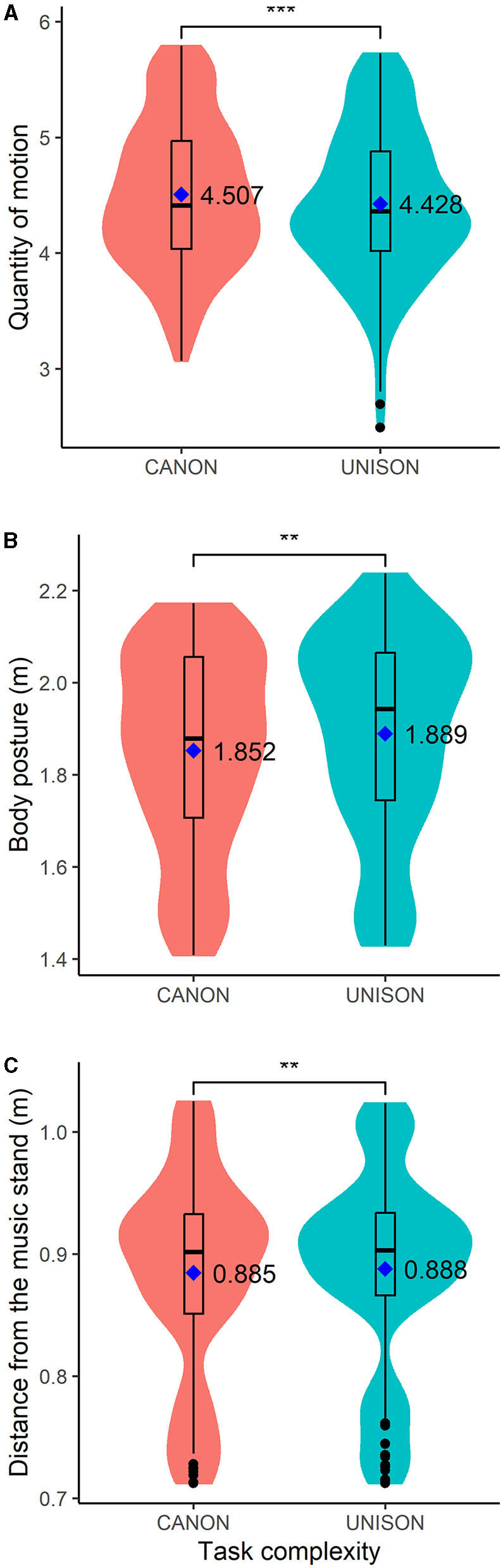
Box plots of motion data related to the quantity of motion **(A)**, body posture **(B)**, and the distance from the head to the music stand **(C)** when singing in canon and in unison. The figure also displays the mean values for each singing condition. ^**^p < 0.01; ^***^p < 0.001.

Table 3—models 1 and 2 exhibit the results of the analysis of the quantity of motion (*QoM*). *QoM* increased in take 2 compared to take 1, and also in phrases 2, 3, and 4 compared to phrase 1. There were no significant differences in *QoM* between block 1 and block 2. *QoM*, averaged across blocks, was not predicted by voice match but by task complexity: *QoM* was lower when singing in unison than canon (as shown in Figure 4A).

**Table 3:** Results of the linear mixed models measuring the relationship between block, take, phrase (phr), voice-matching and task complexity (i.e., the predictors) with the total quantity of motion and the posture (i.e., the response variables).

**Response variable**	**Model *n***	**Predictors**	**Estimate**	**SE**	***t*-value**	**Random effects**
**Quantity of motion**						
	1					Participant, trial
		block	0.03	0.03	0.29	
		take	0.07*	0.02	2.7	
		phr 2 vs. phr 1	0.26***	0.03	9	
		phr 3 vs. phr 1	0.26***	0.03	9	
		phr 4 vs. phr 1	0.34***	0.03	11.7	
	2					Participant, take, phrase
		voice-matching	0.03	0.02	1.43	
		task complexity	−0.09***	0.02	−4.13	
**Posture**						
	3					Participant, trial
		block	−0.02	0.01	−1.4	
		take	−0.007	0.01	−0.06	
		phr 2 vs. phr 1	−0.05**	0.01	−3.33	
		phr 3 vs. phr 1	−0.065***	0.01	−4.72	
		phr 4 vs. phr 1	−0.072***	0.01	−5.24	
	4					Participant, phrase
		voice-matching	−0.01	0.01	−1.07	
		task complexity	0.04*	0.01	0.004	

The table also displays the model number (*n*) and random effects variables (i.e., participant, take, phrase and trial, depending on the model) used in the analysis. The FDR method has been used for adjusted *p*-values. ^*^p < 0.05; ^**^p < 0.01; ^***^p < 0.001.

### 3.3 Posture

Table 3—models 3 and 4 provide the results of the analysis of the posture data. Block and take number did not predict body posture, but the upper body posture was less open in phrases 2, 3, and 4 compared with phrase 1. Body posture, averaged across blocks and takes, was not predicted by voice match, but by task complexity: body posture was more open when singing in unison than in canon (as shown in Figure 4B).

### 3.4 Distance from stand and loudspeaker

Table 4—models 5 and 6 present the results of the analysis of the distance between the singer's head and the music stand. Block and take were not significant predictors of the distance from the head to the stand, but singers were closer to the stand in phrases 2 and 3 than in phrase 1. Voice match did not predict the distance from the stand, but singers were more distant from the stand when singing in unison than in canon (as shown in Figure 4C).

**Table 4:** Results of the linear mixed models measuring the relationship between block, take, phrase (phr), voice-matching and task complexity (i.e., the predictors) with the 3D distance from the head and to the music stand, and from the head to the loudspeaker (i.e., the response variables).

**Response variable**	**Model *n***	**Predictors**	**Estimate**	**SE**	***t*-value**	**Random effects**
**Distance from stand**						
	5					Participant, trial
		block	−0.001	0.002	−0.4	
		take	0.001	0.002	0.7	
		phr 2 vs. phr 1	−0.006*	0.002	−2.85	
		phr 3 vs. phr 1	−0.008**	0.002	−3.63	
		phr 4 vs. phr 1	−0.005	0.002	−2.13	
	6					Participant, phrase
		voice-matching	−0.0003	0.001	−0.2	
		task complexity	0.003*	0.001	2.6	
**Distance from speaker**						
	7					Participant, trial
		block	−0.004	0.004	−1.34	
		take	0.003	0.003	1.38	
		phr 2 vs. phr 1	−0.001	0.002	−0.56	
		phr 3 vs. phr 1	−0.0002	0.002	−0.11	
		phr 4 vs. phr 1	0.0007	0.002	0.32	
	8					Participant
		voice-matching	0.003	0.002	1.42	
		task complexity	0.0002	0.002	0.083	


The table also displays the model number (*n*) and random effects variables (i.e., participant and trial, depending on the model) used in the analysis. The FDR method has been used for adjusted *p*-values. ^*^p < 0.05; ^**^p < 0.01.

Table 4—models 7 and 8 present the results of the analysis of the distance between the singer's head and the loudspeaker. Block, take and phrase numbers were not significant predictors of the distance from the loudspeaker. The latter, averaged across blocks, take and phrase numbers, was not predicted by voice match or task complexity.

### 3.5 Periodicity of wrist and head motion

Tables 5, 6—models 9 to 14 present the results of the analysis of the periodicity of left and right wrist and head motion. Block and take did not predict the periodicity of the right and left wrist and head (see models 9, 11, and 13). The wrists were more periodic in phrases 2 and 4 than in phrase 1; the head was more periodic in phrase 2 than in phrase 1 (see models 9, 11, and 13).

**Table 5:** Results of the linear mixed models measuring the relationship between block, take, phrase (phr), voice-matching and task complexity (i.e., the predictors) with the periodicity of the left (L) and right (R) wrist motion (i.e., the response variables).

**Response variable**	**Model *n***	**Predictors**	**Estimate**	**SE**	***t*-value**	**Random effects**
**L wrist periodicity**						
	9					Participant, trial
		block	−0.1	2.13	−0.05	
		take	1.22	2.15	0.57	
		phr 2 vs. phr 1	7.48*	3	2.49	
		phr 3 vs. phr 1	5.03	3	1.68	
		phr 4 vs. phr 1	9.48**	3	3.16	
	10					Participant, phrase
		voice-matching	−3.3	2.42	−1.36	
		task complexity	5.93*	2.42	2.45	
**R wrist periodicity**						
	11					Participant, trial
		block	0.89	1.97	0.45	
		take	0.91	2	0.46	
		phr 2 vs. phr 1	8.35**	2.78	3.01	
		phr 3 vs. phr 1	5.6	2.78	2.02	
		phr 4 vs. phr 1	10.7***	2.78	3.86	
	12					Participant, phrase
		voice-matching	−2.67	2.3	−1.16	
		task complexity	6.67*	2.3	2.9	


The table also displays the model number (*n*) and random effects variables (i.e., participant, trial and phrase, depending on the model) used in the analysis. The FDR method has been used for adjusted *p*-values. ^*^p < 0.05; ^**^p < 0.01; ^***^p < 0.001.

**Table 6:** Results of the linear mixed models measuring the relationship between block, take, phrase (phr), voice-matching and task complexity (i.e., the predictors) with the periodicity of the head and chest motion (i.e., the response variables).

**Response variable**	**Model *n***	**Predictors**	**Estimate**	**SE**	***t*- value**	**Random effects**
**Head periodicity**						
	13					Participant, trial
		block	−1.6	1.79	−0.89	
		take	1.43	1.82	0.78	
		phr 2 vs. phr 1	9.03**	2.53	3.57	
		phr 3 vs. phr 1	4.46	2.53	1.77	
		phr 4 vs. phr 1	5.68	2.53	2.25	
	14					Participant, take, phrase
		voice-matching	−2.27	2.33	−0.98	
		task complexity	3.32	2.33	1.43	
**Torso periodicity**						
	15					Participant, trial
		block	2.5	2.5	1	
		take	1.2	2.3	0.5	
		phr 2 vs. phr 1	10.8**	3	3.6	
		phr 3 vs. phr 1	9.7**	3	3.2	
		phr 4 vs. phr 1	10.6**	3	3.6	
	16					Participant, phrase
		voice-matching				
		task complexity	6.6*	2.4	2.7	

The table also displays the model number (*n*) and random effects variables (i.e., participant, trial and phrase, depending on the model) used in the analysis. The FDR method has been used for adjusted *p*-values. ^*^p < 0.05; ^**^p < 0.01.

**Figure 5:**
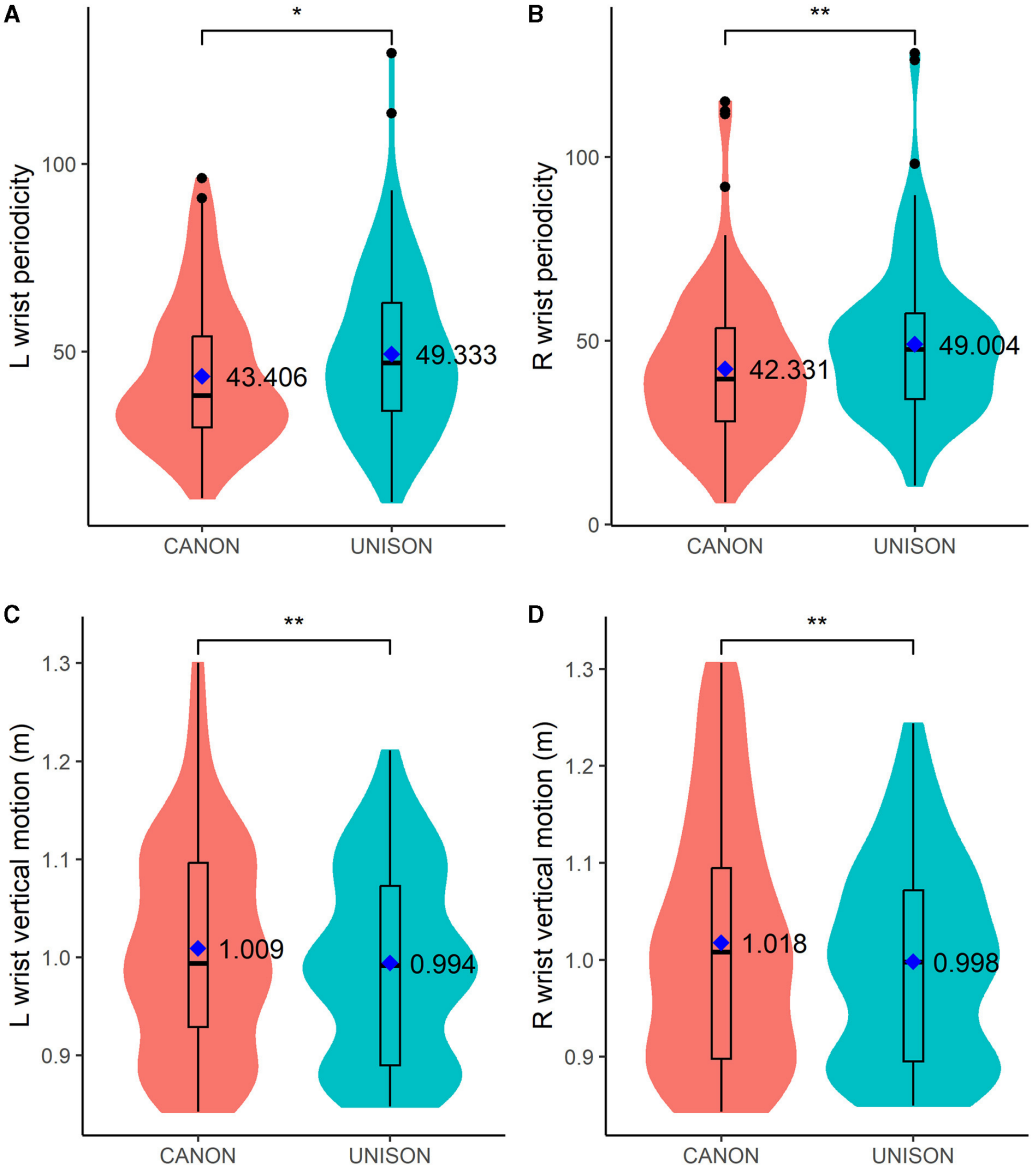
Box plots of motion data related to the left (L) and right (R) wrist periodicity (**A, B**, respectively) and vertical motion (**C, D**, respectively). The figure also displays the mean values for each singing condition and motion parameter. ^*^p < 0.05; ^**^p < 0.01.

Voice match did not predict the periodicity of the wrist or the head, averaged across blocks and takes (see models 10, 12, and 14). The right and left wrists were more periodic when singing in unison than in canon (see Table 5 models 10 and 12 and Figures 5A, B), but task complexity did not predict the periodicity of the head motion, averaged across blocks and takes (see Table 6 model 14).

Having found that task complexity predicted the periodicity of the wrists but not the head motion, it was of interest to investigate whether the wrist motion was related to body sway or not. To test this, we measured the *WT* power of the marker placed on the back top of the singer, similarly to the *WT* power of the head and wrist. We then implemented two linear mixed models (see Table 6 models 15 and 16), based on the same structure of the head and wrist periodicity computation. We found the torso was more periodic when singing in unison than in canon (see Table 6 model 16), in line with the wrists' motion. These results suggest that task complexity predicted singers' body sway.

### 3.6 Wrist vertical motion

Table 7—models 17 to 20 present the results of the analysis of the vertical motion of the right and left wrist, related to the effects of block, take, phrase, voice match and task complexity. Block and take number did not predict the vertical motion of the right and left wrist, but phrase number did: left and right wrists were higher in phrases 2, 3, and 4 compared with phrase 1 (see Table 7 models 17 and 19 and Figures 5C, D). The vertical position of both wrists, aggregated across blocks and takes, was lower when singing in unison than in canon (see models 18 and 20). The position of the right wrist was also higher when singing along with the least together setting than with the most together setting; but, voice match did not predict the vertical position of the left wrist (see models 18 and 20).

**Table 7:** The table also displays the model number (*n*) and random effects variables (i.e., participant and trial, depending on the model) used in the analysis. The FDR method has been used for adjusted *p*-values. ^*^p < 0.05; ^**^p < 0.01; ^***^p < 0.001.

**Response variable**	**Model *n***	**Predictors**	**Estimate**	**SE**	***t*-value**	**Random effects**
**L wrist position**						**Participant, trial**
	17					
		block	0.01	0.01	1.88	
		take	−0.01	0.01	−1.38	
		phr 2 vs. phr 1	0.02*	0.01	2.6	
		phr 3 vs. phr 1	0.02*	0.01	2.55	
		phr 4 vs. phr 1	0.02**	0.01	3.22	
	18					Participant
		voice-matching	0.01	0.01	0.12	
		task complexity	−0.01*	0.01	−2.84	
**R wrist position**						
	19					Participant, trial
		block	−0.01	0.01	−0.61	
		take	0.01	0.01	1.6	
		phr 2 vs. phr 1	0.02**	0.01	3.2	
		phr 3 vs. phr 1	0.03**	0.01	3.67	
		phr 4 vs. phr 1	0.03***	0.01	4.87	
	20					Participant
		voice-matching	0.02*	0.01	2.6	
		task complexity	−0.02**	0.01	−3.02	

Results of the linear mixed models measuring the relationship between block, take, phrase (phr), voice-matching and task complexity (i.e., the predictors) with the vertical position of the right (R) and left (L) wrist (i.e., the response variables).

**Figure 6:**
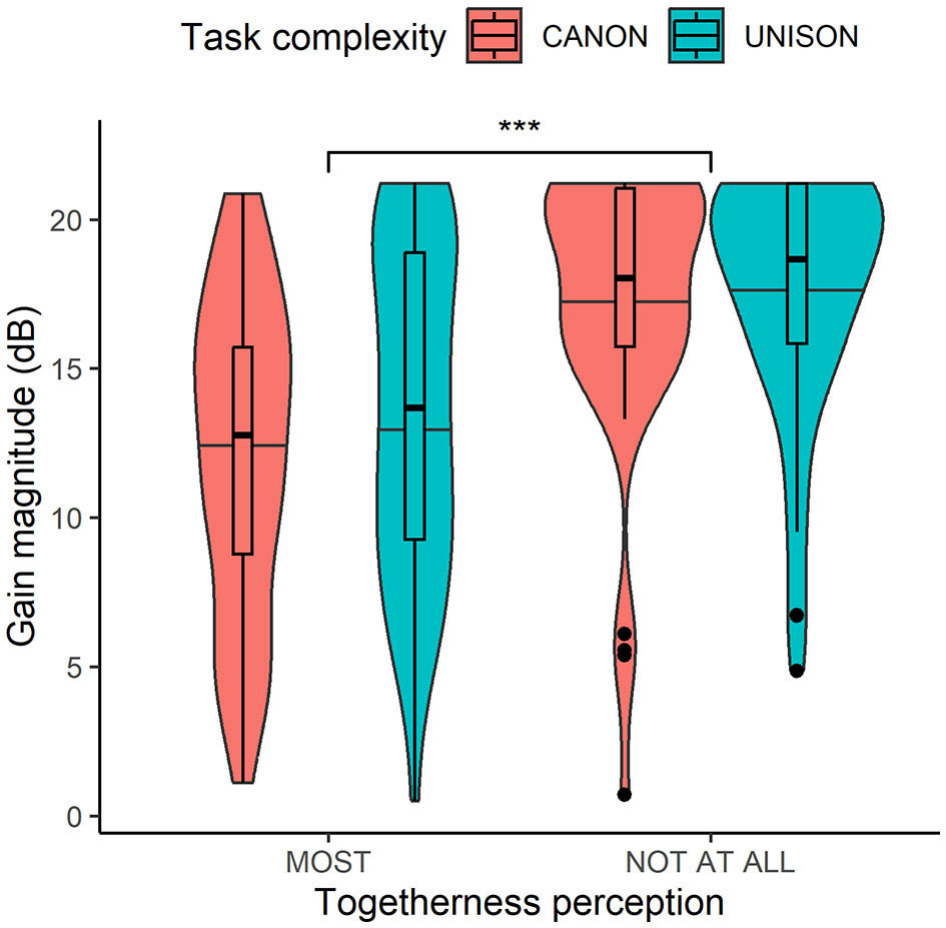
Box plots of the magnitude in dB of the chosen filter settings related to the togetherness perception (i.e., most and not at all together) and task complexity (i.e., singing in unison and in canon). ***p < 0.001.

Interestingly, tuning estimates predicted the wrists' vertical position, depending on block and task complexity. When singing in unison, the higher the pitch, the higher the right wrist in block 1 was (β = 0.004, *p* < 0.005). When singing in canon, the higher the pitch, the higher the left wrist was in block 1 and block 2 (for block 1: (β = 0.002, *p* < 0.001; for block 2:(β = 0.002, *p* < 0.01). We found no evidence of an effect of tuning on the right wrist when singing in unison in block 2 or canon. We also found no evidence of an effect of tuning on the left wrist when singing in unison.

## 4 Discussion

This study focused on choral singers and investigated how body motion is affected by the perceived voice match with a co-performer (as measured based on the sound spectral envelope) and by the complexity of the singing task (such as singing in unison or canon). We were also interested in whether the tuning tracking of the piece performed predicted the continuous vertical displacement of the choristers' wrists. We used a singer-loudspeaker paradigm where choristers performed along to a pre-recorded tune presented over the loudspeaker; this allowed the chorister to manipulate the spectral envelope of the co-performer and identify the settings that they felt most together with and least together with.

Our results revealed that choristers' perceived voice match with the pre-recorded tune was related to the long-term spectrum envelope (hypothesis, H1). The mean magnitude of the high and mid-band filter settings was 13 dB for the recordings judged most together and 17 dB for those identified as least together. This suggests that singers' ratings for the least together performance relate to extreme differences between the spectral components. As shown in Figure 6, some participants appeared to develop a strategy of exploiting the min/max gain color signal in order to find the extreme settings rapidly; and, those settings would often but not always be chosen as “not at all together”. However, given that the spectral manipulations offered were rather subtle and unfamiliar, we found it was helpful to be able to exaggerate the spectral changes, such that it would become obvious which aspect of the sound one was controlling. Furthermore, the extent to which the spectral components are blended might also be related to the choristers' roles and intentions. It has been found that boys with the deepest voices (the basses) boosted the energy of a high-frequency band (2.500–3.500 Hz) of the vocal spectrum when performing in the presence of female listeners, which might be an indication of competitive mechanisms between males (Keller et al., 2017). Future investigations might also investigate the notion of togetherness in relation to the spectral components and the choristers' roles and intentions within the choir.

Contrary to our expectations (H2), we did not find evidence of a significant impact of the chosen voice-matching settings on singers' body motion. The chosen settings did not predict singers' body posture, quantity of motion and head and wrist periodicity, or distance from the speaker. This suggests that voice-matching perception, quantified in terms of spectral components, might be irrelevant to body motion. However, it might be that these models lacked the necessary power to find the effect. Future investigations with a larger sample size and power tests might provide comprehensive results on the null effects of voice matching on body motion that we observed in this study. It might also be that the singer-loudspeaker paradigm we implemented in this study did not capture the interpersonal dynamics of a singing duo. A recent case study analyzing music interactions in virtual reality reported that performing in a piano duo with a computer-controlled agent can be inadequate for the musicians' subjective experience (Van Kerrebroeck et al., 2021). Authors found that a professional pianist reported lower scores on enjoyment, closeness, and naturalness when performing with a controlled agent (i.e., in which an algorithm aligned the audio-visual information of an avatar to the real-time performance of the pianist) than in duo with a virtual avatar (i.e., a pianist visually perceiving the co-performer as a human-embodied virtual avatar). Future empirical investigations with a larger sample size are needed to analyse the impact of non-human entities on musicians' immersion in ensemble performances. The absence of an audience might also have underestimated the effects of the sensation of togetherness on body motion. The increased level of immersion of a real public performance might emphasize the impact of togetherness on musicians' body motion.

In line with our expectations, we found that singing the same tune in unison or canon (i.e., with a temporal delay) affected body motion in several ways (H3). First, the quantity of motion was higher when singing in canon, in line with the overall increased energy of the performance (H3.1). Then, choristers were closer to the music stand when singing in canon (H3.2): this suggests the increased complexity of the canon task and might reflect the need to be closer to the music to see more clearly and cut out distractions by reducing the visual field. However, standing closer to the music stand does not necessarily indicate that the singers were looking at the score. Future analysis investigating eye-gaze might shed more light in this respect. Furthermore, the motion of both wrists were more periodic when singing in unison than canon (H3.3). These results can be interpreted as a disruptive mechanism in the auditory feedback affecting hand motion: during canon singing, hearing the pre-recorded tune as auditory feedback—serially shifted relative to their own tones—might have disrupted the periodicity of the hand motion. This hypothesis is in line with research suggesting that music performance is disrupted, i.e., increased errors of sequencing and timing, when the auditory feedback of actions is disrupted (Pfordresher and Palmer, 2006; Palmer et al., 2019). It is also interesting to notice that the higher periodicity of the wrists was paired with that of the torso, suggesting that the unison singing promotes upper body swaying. This might be due to the ease of the task complexity and/or to the fact the singers might feel more integrated when singing in unison. Their body posture was more open when singing in unison than canon: singers might have felt more open to communication whilst performing the easier task, which might have manifested in a more open body posture in the unison than canon task.

Interestingly, we did not find an effect of voice-matching perception or task complexity on singers' head motion. The latter is often associated with visual expressivity (Glowinski et al., 2013; Bishop et al., 2019) and was found to be more variable when singing in canon than unison (Palmer et al., 2019). More recently, it has been found that the similarity in common periodic oscillations of musicians' head motions was related to the musicians' empathic profile and the phrase structure of the piece (D'Amario et al., 2023). It has also been found that musicians' head motion in ensembles can change under conditions that require more self-regulation, suggesting that head motion, in addition to communicative functions, can support regulation of the own performance (Laroche et al., 2022). In the current study, the lack of a real co-performer and an audience might have underestimated its role. Future ecologically valid investigations involving real singing ensembles might test whether musicians' perception of voice matching with a co-performer and the singing task impact their head motion.

We found that the vertical position of the wrists was positively related to the performed pitch (H4). These results corroborate the literature revealing a coupling between singers' arm gestures and intonation (Brunkan, 2015; Brunkan and Bowers, 2021; Pearson and Pouw, 2022). Our findings are in line with the sound-tracing experiments suggesting that hand motion is a spatial representation of space or the pitch order (Godøy et al., 2006; Nymoen et al., 2010, 2011, 2013). This positive correlation could also be understood in light of the increased difficulty in tuning higher notes, which singers anecdotally report. The fact that we found evidence of this correlation mostly in the most difficult performance contexts, i.e., when singing in canon and when singing in unison for the first times, suggests that singers' hand motion, although not strictly linked to the sound producing, might fulfill musical purposes, by coming into play to facilitate the performance context. These findings also expand the literature analyzing hand motion of instrumental musicians, which suggests that certain hand and finger movements, such as increased movement amplitude, facilitate faster tempi in piano performance (Palmer and Dalla Bella, 2004).

We also found that the right wrist position was associated with tuning in the unison performance, whilst the left wrist was related to tuning in the canon performance. This might indicate that the left hand supports the most difficult context performance; however, this might also depend on the singer being left- or right-handed, an aspect not assessed in this study. Future investigations might shed more light in this respect to evaluate whether the left hand plays a technical supporting role stronger than the right hand. We also found that when singing along with the least-together performance, the right wrist position was higher than that singers had when singing with the most-together setting. This result can be understood in light of the previous results regarding task complexity and tuning: the wrist's vertical position seems to play a fundamental role in singing by supporting tuning of higher pitch (anecdotally considered more difficult than the lower ones) and performance in the most difficult context, such as singing in canon vs. unison and along the least than most together setting.

## 5 Limitations

At the beginning of the experiment, most of the participants self-reported past experiences of high levels of togetherness feelings with a co-performer. During moments of optimal matching experienced in the past, they felt as if their voices became *one* and everything was *locked in* and *flow*, as shown in Table 2. These experiences are in line with many dimensions of individual and group flow (Csikszentmihalyi, 1996). Importantly, these self-reported experiences demonstrate that our participants were familiar with the concepts of togetherness feelings and optimal matching. Nevertheless, it remains unknown whether our participants experienced feelings of togetherness *during* our production task. Future follow-up investigations could investigate continuous ratings of togetherness feelings in singing performances, resulting from the manipulation of the sound spectral envelope, to investigate the relationship between sound envelope and togetherness. It also remains unknown how their perceived optimal match (conceptualized in terms of spectral components) relates to the broad range of togetherness feelings (resulting from the social and cognitive alignment with the co-performer that varies as the music unfolds (Bishop, 2023; D'Amario et al., 2023). Future studies based on self-reported experiences might shed more light in this respect by investigating how musicians conceptualize togetherness feelings and voice match sensation.

## 6 Conclusion

By adopting a singer-loudspeaker paradigm, this study showed that the sound spectral envelope affects the sensation of being together with a pre-recorded voice. However, the present results suggest that voice-matching preferences do not make a major impact on choristers' body motion. The study also revealed that many aspects of the choristers' body motions relate to the complexity of the singing task (i.e., singing in unison or canon). Interestingly, wrist motion responded to togetherness perception, task complexity and tuning, revealing its importance in singing. These results can be of interest to choir and solo singing pedagogy aiming at identifying strategies for performance excellence.

## Data availability statement

The datasets presented in this study can be found at https://doi.org/10.5281/zenodo.8380830.

## Ethics statement

The studies involving humans were approved by Ethics Committee at mdw - University of Music and Performing Arts Vienna. The studies were conducted in accordance with the local legislation and institutional requirements. The participants provided their written informed consent to participate in this study. Written informed consent was obtained from the individual(s) for the publication of any potentially identifiable images or data included in this article.

## Author contributions

SD and ST equally contributed to the conception and design of the study. SD was the main contributor to motion data acquisition, analysis and interpretation and drafted the article. ST made a substantial contribution to audio data acquisition. ST, LB, and WG contributed to analyzing and interpreting the data collected. All authors critically revised the article and approved the submitted version.

## References

[B1] Albin A. L. (2014). PraatR: an architecture for controlling the phonetics software “Praat” with the R programming language. J. Acoust. Soc. Am. 135, 2198. 10.1121/1.4877175

[B2] Apfelstadt H. (1985). Choral music in motion: the use of movement in the choral rehearsal. Choral J. 25, 37–39.

[B3] Bates D. Machler M. Bolker B. Walker S. (2015). Fitting linear mixed-effects models using lme4. J. Stat. Softw. 67, 1–48. 10.18637/jss.v067.i01

[B4] Benjamini Y. Hochberg Y. (1995). Controlling the false discovery rate: a practical and powerful approach to multiple testing. J. R. Stat. Soc. Ser. B Stat. Methodol. 57, 289–300.

[B5] Bishop L. (2023). Focus of attention affects togetherness experiences and body interactivity in piano duos. Psychol. Aesthet. Creat. Arts. 10.1037/aca0000555

[B6] Bishop L. Cancino-Chacon C. Goebl W. (2019). Moving to communicate, moving to interact. Music Percept. 37, 1–25. 10.1525/mp.2019.37.1.1

[B7] Brooks M. E. Kristensen K. van Benthem K. J. Magnusson A. Berg C. W.. (2017). glmmTMB balances speed and flexibility among packages for zero-inflated generalized linear mixed modeling. R J. 9, 378–400. 10.32614/RJ-2017-066

[B8] Brunkan D. M. Bowers D. J. (2021). Singing with gesture: acoustic and perceptual measures of solo singers. J. Voice 35, 325.e17–325.e22. 10.1016/j.jvoice.2019.08.029 31607422

[B9] Brunkan M. C. (2015). The effects of three singer gestures on acoustic and perceptual measures of solo singing. Int. J. Music Perform. Arts 3, 35–45. 10.15640/ijmpa.v3n1a4

[B10] Brunkan M. C. (2016). Relationships of a circular singer arm gesture to acoustical and perceptual measures of singing. Update Appl. Res. Music Educ. 34, 56–62. 10.1177/8755123314567782

[B11] Clift S. Hancox G. Morrison I. Hess B. Kreutz G. Stewart D. (2010). Choral singing and psychological wellbeing: quantitative and qualitative findings from English choirs in a cross-national survey. J. Appl. Arts Health 1, 19–34. 10.1386/jaah.1.1.19/1

[B12] Csikszentmihalyi M. (1996). The Flow of Creativity. New York, NY: Harper Collins.

[B13] Daffern H. Balmer K. Brereton J. (2021). Singing together, yet apart: the experience of UK choir members and facilitators during the COVID-19 pandemic. Front. Psychol. 12, 624474. 10.3389/fpsyg.2021.624474 33679542 PMC7930073

[B14] D'Amario S. Daffern H. (2017). Using electrolaryngography and electroglottography to assess the singing voice: a systematic review. Psychomusicol. Music Mind Brain 27, 229–243. 10.1037/pmu0000184

[B15] D'Amario S. Goebl W. Bishop L. (2022). Judgment of togetherness in performances by musical duos. Front. Psychol. 13, 997752. 10.3389/fpsyg.2022.997752 36467141 PMC9716625

[B16] D'Amario S. Schmidbauer H. Roesch A. Goebl W. Niemand A. Bishop L. (2023). Interperformer coordination in piano-singing duo performances: phrase structure and empathy impact. Psychol. Res. 87, 2559–2582. 10.1007/s00426-023-01818-8 37074403 PMC10497663

[B17] Daugherty J. F. Grady M. L. Coffeen R. C. (2019). Effects of choir spacing and riser step heights on acoustic and perceptual measures of SATB choir sound acquired from four microphone positions in two performance halls. J. Res. Music Educ. 67, 355–371. 10.1177/0022429419844508

[B18] Daugherty J. F. Manternach J. N. Brunkan M. C. (2012). Acoustic and perceptual measures of SATB choir performances on two types of portable choral riser units in three singer-spacing conditions. Int. J. Music Educ. 31, 359–375. 10.1177/0255761411434499

[B19] Davidson J. W. (2001). The role of the body in the production and perception of solo vocal performance: a case study of annie lennox. Music. Sci. 5, 235–256. 10.1177/102986490100500206

[B20] Dell'Anna A. Rosso M. Bruno V. Garbarini F. Leman M. Berti A. (2020). Does musical interaction in a Jazz duet modulate peripersonal space? Psychol. Res. 85, 2107–2118. 10.1007/s00426-020-01365-6 32488599

[B21] Eerola T. Jakubowski K. Moran N. Keller P. E. Clayton M. (2018). Shared periodic performer movements coordinate interactions in duo improvisations. R. Soc. Open Sci. 5, 171520. 10.1098/rsos.171520 29515867 PMC5830756

[B22] Eitan Z. Granot R. Y. (2006). How music moves. Music Percept. 23, 221–248. 10.1525/mp.2006.23.3.221

[B23] Gilliam T. M. (2020). Effects of three common choral configurations on acoustic and perceptual measures of a soprano section's sound. Update Appl. Res. Music Educ. 39, 54–66. 10.1177/8755123320965935

[B24] Glowinski D. Mancini M. Cowie R. Camurri A. Chiorri C. Doherty C. (2013). The movements made by performers in a skilled quartet: a distinctive pattern, and the function that it serves. Front. Psychol. 4, 841. 10.3389/fpsyg.2013.00841 24312065 PMC3826428

[B25] Godøy R. I. Haga E. Jensenius A. R. (2006). “Exploring music-related gestures by sound-tracing. a preliminary study,” in 2nd ConGAS International Symposium on Gesture Interfaces for Multimedia Systems (Leeds).

[B26] Grachten M. Demey M. Moelants D. Leman M. (2010). “Analysis and automatic annotation of singer's postures during concert and rehearsal,” in Proceeding of the 7th Sound and Music Computing Conference (Barcelona), 191–198.

[B27] Hart Y. Noy L. Feniger-Schaal R. Mayo A. E. Alon U. (2014). Individuality and togetherness in joint improvised motion. PLoS ONE 9, e87213. 10.1371/journal.pone.0087213 24533054 PMC3922750

[B28] Herbst C. T. (2020). Electroglottography–an update. J. Voice 34, 503–526. 10.1016/j.jvoice.2018.12.014 30871855

[B29] Himberg T. Laroche J. Bige R. Buchkowski M. Bachrach A. (2018). Coordinated interpersonal behaviour in collective dance improvisation: the aesthetics of kinaesthetic togetherness. Behav. Sci. (Basel). 8, 23. 10.3390/bs8020023 29425178 PMC5836006

[B30] Himberg T. Thompson M. (2009). “Group synchronization of coordinated movements in a cross-cultural choir workshop,” in Proceedings of the 7th Triennal Conference of European Society for Cognitive Sciences of Music (ESCOM 2009) eds J. Louhivuori, S. Eerola, S. Saarikallio, T. Himberg, and P. S. Eerola (Jyväskylä), 175–180.

[B31] Jakubowski K. Eerola T. Ximenes A. B. Ma W. K. Clayton M. Keller P. E. (2020). Multimodal perception of interpersonal synchrony: evidence from global and continuous ratings of improvised musical duo performances. Psychomusicol. Music Mind Brain 30, 159–177. 10.1037/pmu0000264

[B32] Jensenius A. R. Wanderley M. M. Godøy R. Leman M. (2010). “Musical gestures: concepts and methods in research,” in Musical Gestures: Sound, Movement, and Meaning, R. Godøy and M. Leman (New York, NY: Routledge), 12–35.

[B33] Judd M. Pooley J. A. (2014). The psychological benefits of participating in group singing for members of the general public. Psychol. Music 42, 269–283. 10.1177/0305735612471237

[B34] Kalin G. (2005). Formant frequency adjustment in barbershop quartet singing (Master's thesis). Dept of Speech, Music and Hearing, KTH Royal Institute of Technology, Stockholm, Sweden.

[B35] Keller P. E. Appel M. (2010). Individual differences, auditory imagery, and the coordination of body movements and sounds in musical ensembles. Music Percept. 28, 27–46. 10.1525/mp.2010.28.1.27

[B36] Keller P. E. Konig R. Novembre G. (2017). Simultaneous cooperation and competition in the evolution of musical behavior: sex-related modulations of the singer's formant in human chorusing. Front. Psychol. 8, 1559. 10.3389/fpsyg.2017.01559 28959222 PMC5603663

[B37] Kuznetsova A. Brockhoff P. B. Christensen R. H. B. (2017). lmerTest package: tests in linear mixed effects models. J. Stat. Softw. 82, 1–26. 10.18637/jss.v082.i13

[B38] Laroche J. Tomassini A. Volpe G. Camurri A. Fadiga L. D'Ausilio A. (2022). Interpersonal sensorimotor communication shapes intrapersonal coordination in a musical ensemble. Front. Hum. Neurosci. 16, 899676. 10.3389/fnhum.2022.899676 36248684 PMC9556642

[B39] Leman M. (2007). Embodied Music Cognition and Mediation Technology. Cambridge, MA: MIT Press.

[B40] Livesey L. Morrison I. Clift S. Camic P. (2012). Benefits of choral singing for social and mental wellbeing: qualitative findings from a cross-national survey of choir members. J. Public Mental Health 11, 10–26. 10.1108/17465721211207275

[B41] Livingstone S. R. Palmer C. (2016). Head movements encode emotions during speech and song. Emotion 16, 365–380. 10.1037/emo0000106 26501928

[B42] Livingstone S. R. Thompson W. F. Russo F. A. (2009). Facial expressions and emotional singing: a study of perception and production with motion capture and electromyography. Music Percept. 26, 475–488. 10.1525/mp.2009.26.5.475

[B43] McGinley H. LeFevre R. McGinley P. (1975). The influence of a communicator's body position on opinion change in others. J. Pers. Soc. Psychol. 31, 686–690.

[B44] Nafisi J. (2013). Gesture and body-movement as teaching and learning tools in the classical voice lesson: a survey into current practice. Brit. J. Music Educ. 30, 347–367. 10.1017/S0265051712000551

[B45] Noy L. Levit-Binun N. Golland Y. (2015). Being in the zone: physiological markers of togetherness in joint improvisation. Front. Hum. Neurosci. 9, 187. 10.3389/fnhum.2015.00187 25999832 PMC4419713

[B46] Nymoen K. Caramiaux B. Kozak M. Torresen J. (2011). “Analyzing sound tracings: a multimodal approach to music information retrieval,” in Proceedings of the 1st International ACMworkshop on Music Information Retrieval with User-Centered and Multimodal Strategies. MIRUM '11 (New York, NY: ACM), 39–44.

[B47] Nymoen K. Glette K. Skogstad S. A. Torresen J. Jensenius A. R. (2010). “Searching for cross-individual relationships between sound and movement features using an SVM classifier,” in Proceedings of the International Conference on New Interfaces for Musical Expression (Sydney, NSW), 259–262.

[B48] Nymoen K. Godøy R. I. Jensenius A. R. Torresen J. (2013). Analyzing correspondence between sound objects and body motion. ACM Trans. Appl. Percept. 10, 1–22. 10.1145/2465780.2465783

[B49] Palmer C. Dalla Bella S. (2004). Movement amplitude and tempo change in piano performance. J. Acoust. Soc. Am. 115, 2590–2590. 10.1121/1.4784407

[B50] Palmer C. Spidle F. Koopmans E. Schubert P. (2019). Ears, heads, and eyes: when singers synchronise. Q. J. Exp. Psychol. 72, 2272–2287. 10.1177/1747021819833968 30744490

[B51] Pearson L. Pouw W. (2022). Gesture-vocal coupling in karnatak music performance: a neuro-bodily distributed aesthetic entanglement. Ann. N. Y. Acad. Sci. 1515, 219–236. 10.1111/nyas.14806 35730069

[B52] Peterson C. W. (2000). Moving musical experiences in chorus. Music Educ. J. 86, 28–30. 10.2307/3399617

[B53] Pfordresher P. Q. (2005). Auditory feedback in music performance: the role of melodic structure and musical skill. J. Exp. Psychol. Hum. Percept. Perform. 31, 1331–1345. 10.1037/0096-1523.31.6.1331 16366793

[B54] Pfordresher P. Q. Palmer C. (2006). Effects of hearing the past, present, or future during music performance. Percept. Psychophys. 68, 362–376. 10.3758/BF03193683 16900830

[B55] R Core Team (2013). R: A Language and Environment for Statistical Computing. Vienna: R Foundation for Statistical Computing.

[B56] Repp B. H. (1992). Diversity and commonality in music performance: an analysis of timing microstructure in Schumann's “Träumerei”. J. Acoust. Soc. Am. 92, 2546–2568. 1479119 10.1121/1.404425

[B57] Roesch A. Schmidbauer H. (2018). Waveletcomp: Computational Wavelet Analysis. R package version 1.1.

[B58] Stevens A. Ramirez-Lopez L. (2021). An Introduction to the Prospectr Package. R package version 0.2.2.

[B59] Ternström S. (1993). Perceptual evaluations of voice scatter in unison choir sounds. J. Voice 7, 129–135. 8353626 10.1016/s0892-1997(05)80342-x

[B60] Timmers R. Endo S. Bradbury A. Wing A. M. (2014). Synchronization and leadership in string quartet performance: a case study of auditory and visual cues. Front. Psychol. 5, 645. 10.3389/fpsyg.2014.00645 25002856 PMC4066619

[B61] Van Kerrebroeck B. Caruso G. Maes P. J. (2021). A methodological framework for assessing social presence in music interactions in virtual reality. Front. Psychol. 12, 663725. 10.3389/fpsyg.2021.663725 34177720 PMC8226187

[B62] Vicary S. Sperling M. von Zimmermann J. Richardson D. C. Orgs G. (2017). Joint action aesthetics. PLoS ONE 12, e0180101. 10.1371/journal.pone.0180101 28742849 PMC5526561

[B63] von Zimmermann J. Vicary S. Sperling M. Orgs G. Richardson D. C. (2018). The choreography of group affiliation. Top. Cogn. Sci. 10, 80–94. 10.1111/tops.12320 29327424 PMC6092630

[B64] Zamm A. Pfordresher P. Q. Palmer C. (2015). Temporal coordination in joint music performance: effects of endogenous rhythms and auditory feedback. Exp. Brain Res. 233, 607–615. 10.1007/s00221-014-4140-5 25399244 PMC4295031

